# Fractional carbon dioxide laser assisted delivery of tranexamic acid versus ascorbic acid in the treatment of melasma: a split face comparative study with digital skin analysis

**DOI:** 10.1007/s10103-025-04529-1

**Published:** 2025-06-16

**Authors:** Bayoumy I. Eassa, Amr M. Ammar, Omar A. Shoaib, Mohamed L. Elsaie

**Affiliations:** 1 Al Azhar University, Cairo, Egypt; 2 National Research Centre, Cairo, Egypt

**Keywords:** Melasma, Skin, Pigmentary disorders, Laser

## Abstract

**Purpose:**

Melasma is a chronic, acquired disorder of focal hypermelanosis. It clinically presents as hyperpigmented patches over photo-exposed areas such as the face. The study aimed to evaluate the efficacy and safety of combined fractional CO2 laser with topical tranexamic acid (TA) versus combined fractional CO2 laser with topical ascorbic acid in treating patients with melasma.

**Methods:**

Forty individuals, spanning the ages of 20 to 50, diagnosed with melasma were included in this prospective split-face comparison study. Patients were subjected to detailed medical, skin, dermoscopic, and Woods light, digital skin assessment for their grading of melasma before and after treatment. Subjects received fractional CO2 laser sessions to the affected areas before applying 10% solution of TA on the right side and 20% ascorbic acid solution on the left side.

**Results:**

Baseline dermoscopic examination of the right side showed that 62.5% of the patients had fading of pseudo reticular network, 22.5% had fading telangiectasia, while the other 15% had no change. Considering the left side, 55% had fading of pseudo reticular network, 40% had fading telangiectasia, while the other 5% had no change. The baseline characteristics of melasma were comparable except for vascularity which was significantly higher in the right side compared to the left (*p* = 0.007).

**Conclusion:**

Trans-epidermal delivery of tranexamic acid or vitamin C after low power fractional CO2 laser is a useful tool with more superiority towards the tranexamic acid treatment. More controlled larger sample trials are required to establish an optimal and effective treatment for melasma.

**Graphical abstract:**

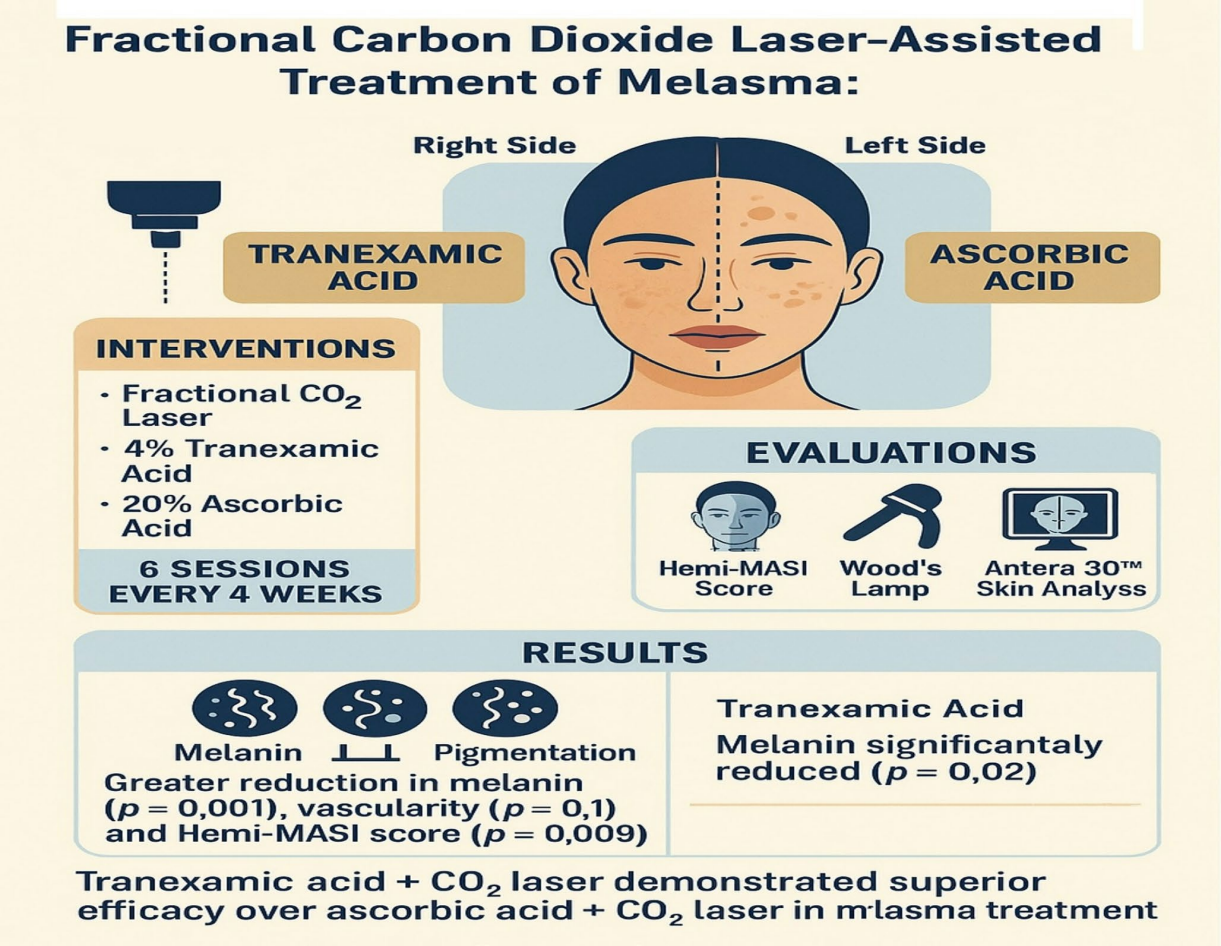

## Introduction

Melasma is a chronic, acquired disorder of focal hypermelanosis. It clinically presents as hyperpigmented patches over photo-exposed areas such as the face. It is more common in, but not exclusive to, women and people with darker skin tones, and can be associated with cumulative ultraviolet (UV) light exposure, pregnancy, heat exposure, contraceptive pill use, and familial predisposition [1]. 

Melasma is a challenging condition to treat with a tendency to recur and can negatively impact the psychological well being of patients. The range of treatments (topical, systemic, and energy-based) for melasma reflects its complex and multifactorial pathogenesis [2]. 

Tranexamic acid (TA) decreases UV-induced pigmentation and blocks the synthesis of melanin in melanocytes. Moreover; the vascular element in melasma induced by the vascular endothelial growth factor (VEGF) as well as angiogenesis which is induced by basic fibroblast growth factor (b-FGF) are reduced by the effects of TA [3]. 

Vitamin C (ascorbic acid) is an abundant antioxidant with antiaging and antioxidant properties. Moreover; vitamin C exerts antipigmentary properties via targeting overactive melanocytes and inhibiting tyrosinase enzyme as well as reducing the oxidation of dopaquinone [4]. 

Laser and light therapies may be utilized cautiously as second line or third-line options for recalcitrant melasma; however, low-energy settings are preferred due to the risk of post-inflammatory hyperpigmentation and melasma stimulation. Lasers can improve the delivery of topical treatments to the target site, as well as work synergistically [5]. 

The aim of this study was to evaluate the efficacy and safety of combined fractional CO2 laser with topical TA versus combined fractional CO2 laser with topical ascorbic acid in treating patients with melasma.

## Patients and methods

Forty individuals, spanning the ages of 20 to 50, diagnosed with melasma were included in this prospective split-face comparison study. Patients were enrolled from the dermatology outpatient clinics at Al-Azhar University Hospitals. The study protocol was approved by the Medical Ethics Committee of Faculty of Medicine, Al-Azhar University (Der_Med 93 Med Research). This study was performed in accordance with the Declaration of Helsinki. All patients signed both Informed Consent Form and Consent for Image Use.

### Inclusion criteria

Patients with melasma of different types, aged 20–50 years, and of both sexes were included in the study.

### Exclusion criteria

Patients on other lines of treatment as topical Kligman’s formula, topical hydroquinone or Q-Switched Nd: YAG Laser sessions, pregnant and lactating women, patients with endocrinal problems causing hyperpigmentation of the face as Addison’s disease, active infection or inflammation of the face, patients with positive history of hypersensitivity reaction to ascorbic acid or tranexamic acid, female patients with menstrual disturbance or ovarian tumors, patients who fail to fulfill the inclusion criteria.

### Methods

A detailed demographic profile and thorough clinical history with details such as the duration, risk factors, and history of treatment received were taken. This was followed by a complete dermatological examination and all findings and information was documented. Baseline and monthly clinical photographs were captured using a Canon EOS750D digital camera. The clinical type of melasma was determined and recorded through clinical assessment and inspection. They were then subjected to examination by the Woods lamp and Dermoscope. The participants were classified into epidermal, dermal and mixed pattern of melasma based on the amount of enhancement noted by the woods lamp. It was classified as epidermal when enhancement was noticed, dermal when there was no enhancement and mixed when there was a slight enhancement noted. Dermoscopy was done at baseline and on monthly basis during the study. A Dermlite DL4 dermoscope was utilized in the study. The color of melanin as well as the intensity and the pattern of pigment network were used to identify the location of pigment. Through the dermoscope the visualization of black and dark brown to light brown color with a regular well defined pigment network was classified as epidermal while the presence of bluish or bluish grey color with an irregular ill-defined pigment network was classified as dermal while combination was classified as mixed.

### Hemi-melasma area and severity index (hemi-MASI) score

Melasma severity was evaluated by two skilled dermatologists independently at baseline and on monthly basis. It was calculated based on area of involvement (A) (0 = 0%, 1 = 1–9%, 2 = 10–29%, 3 = 30–49%, 4 = 50–69%, 5 = 70–89%, 6 = 90–100%); darkness (D) (0 = normal skin color, 1 = slight hyperpigmentation, 2 = mild visible hyperpigmentation, 3 = marked hyperpigmentation, 4 = severe hyperpigmentation); and homogenecity (H) (0 = minimal, 1 = slight, 2 = mild, 3 = marked, 4 = severe) on each side of the face. Thus, the hemi-MASI score = forehead, 0.15 × (D + H) × A + malar, 0.30 × (D + H) × A + chin, 0.05 × (D + H) × A.

### Evaluation by the skin analysis camera system (Antera 3D™, Ireland)

The skin analysis camera system (Antera 3D™, Ireland) was used for objective evaluation. This system makes use of light-emitting diodes of various wavelengths, which are partially absorbed, scattered, and reflected by the skin. The multispectral analysis program allows it to measure the redness, amount of hemoglobin, pigmentation, indentations, wrinkles, and overall roughness of the skin. It can also estimate the uniformity of the melanin distribution. Melasma sites were anatomically matched before and after treatment and measurements were then applied. The results were represented in a percentage that was subsequently used in statistical analysis [6]. All clinical evaluations, including hemi-MASI scoring, dermoscopy, and Antera 3D™ assessments, were performed by evaluators blinded to side allocation.

### Details of the sessions

All patients received the fractional CO2 (BX300; 10,600 nm, Laser Power: 1–40 W, Power Input and Frequency: 220–240 V~, 50/60 Hz, AMI Korea; fluence 15 mJ/cm², density 150 MTZ/cm², spot size 120 μm, pulse duration 600 µs, and one pass per session) laser session to the affected areas before under-occlusion applying 10% solution of TA (100 mg/ml) (Kapron, Amoun Pharmaceutical Co) on the right side and 20% solution ascorbic acid (200 mg/ml) under on the left side. Sessions were repeated every four weeks and for 6 sessions in total. All patients were advised to avoid direct sun exposure with strict sunscreen application before sun exposure by sunscreen with SPF 50.

### Assessment and follow-up

For a duration of six months, patients underwent clinical examinations and had post-procedure photos taken once a month. Results of the treatment were evaluated using the hemi MASI scoring system, dermoscopy, and Wood’s lamp test. Furthermore, unbiased assessment was carried out using the Antera 3DTM skin analysis camera. Any adverse effects from the fractional CO2 laser or drug application were noted.

### Statistical analysis

With the help of SPSS 26 for Windows (SPSS Inc., Chicago, IL, USA), we were able to compile, organize, and analyze all of the data. The Shapiro-Whitney U test was used to ensure that the data followed a normal distribution. Frequencies and relative percentages were used to represent the qualitative data. In order to determine the difference between the qualitative variables, the chi-square test (χ2) and Fisher exact were employed. Parametric data was presented as mean ± SD (Standard deviation), whereas non-parametric data was presented as median and range. A post hoc power analysis indicated that the study had 80% power to detect a 15% difference in hemi-MASI scores between treatment sides, assuming a medium effect size (Cohen’s d = 0.68) and a two-tailed alpha of 0.05. However, no adjustment for multiple comparisons was applied.

## Results

**Fig. 1:**
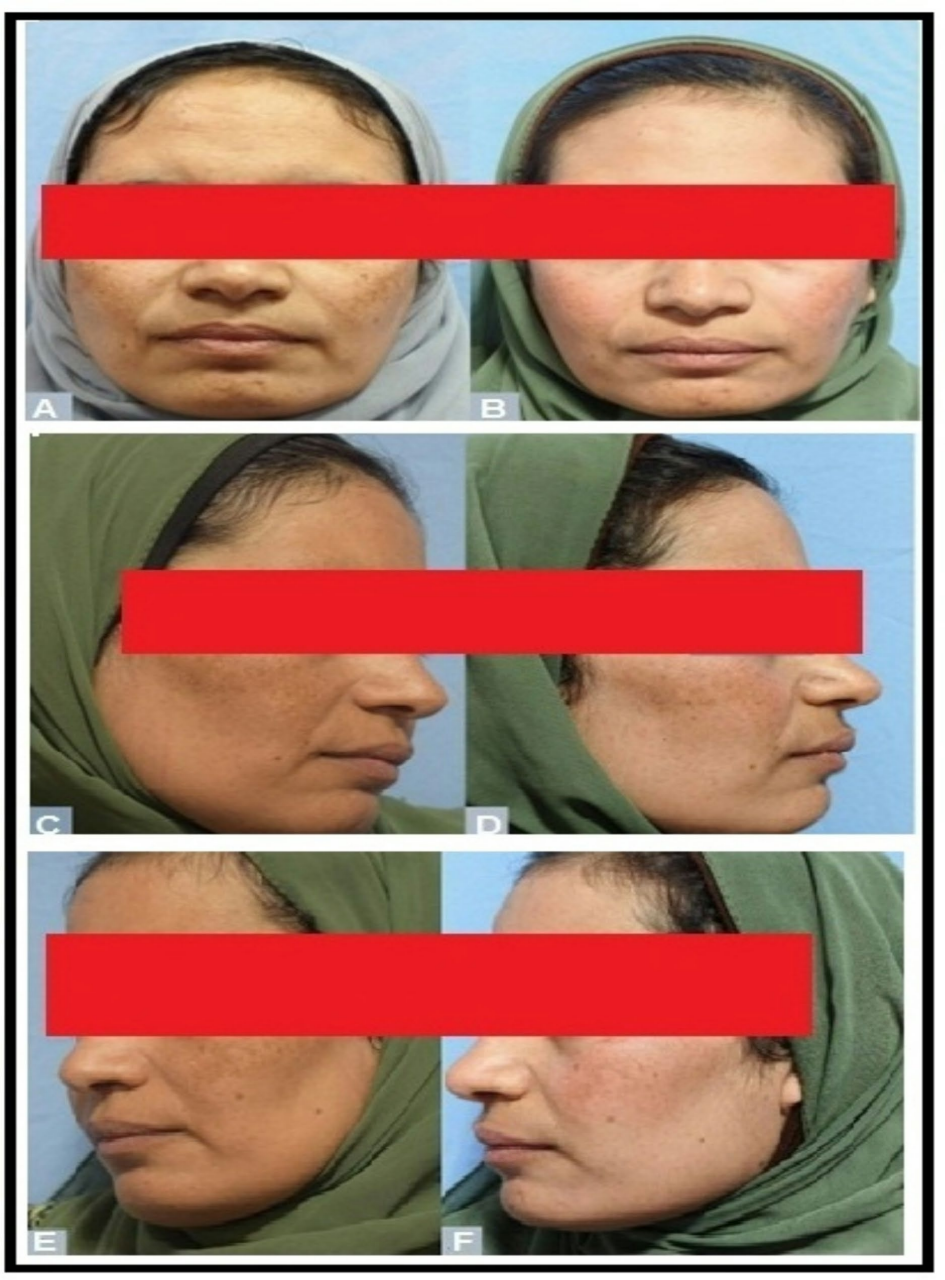
Clinical photographs of a 38-year-old female with melasma before and after treatment. (**A**) Right side before treatment with tranexamic acid. (**B**) Right side after treatment. (**C**) Left side before treatment with ascorbic acid. (**D**) Left side after treatment. (**E**, **F**) Frontal view before and after treatment. Photographs show marked improvement in pigmentation, more evident on the right (TA-treated) side

**Fig. 2:**
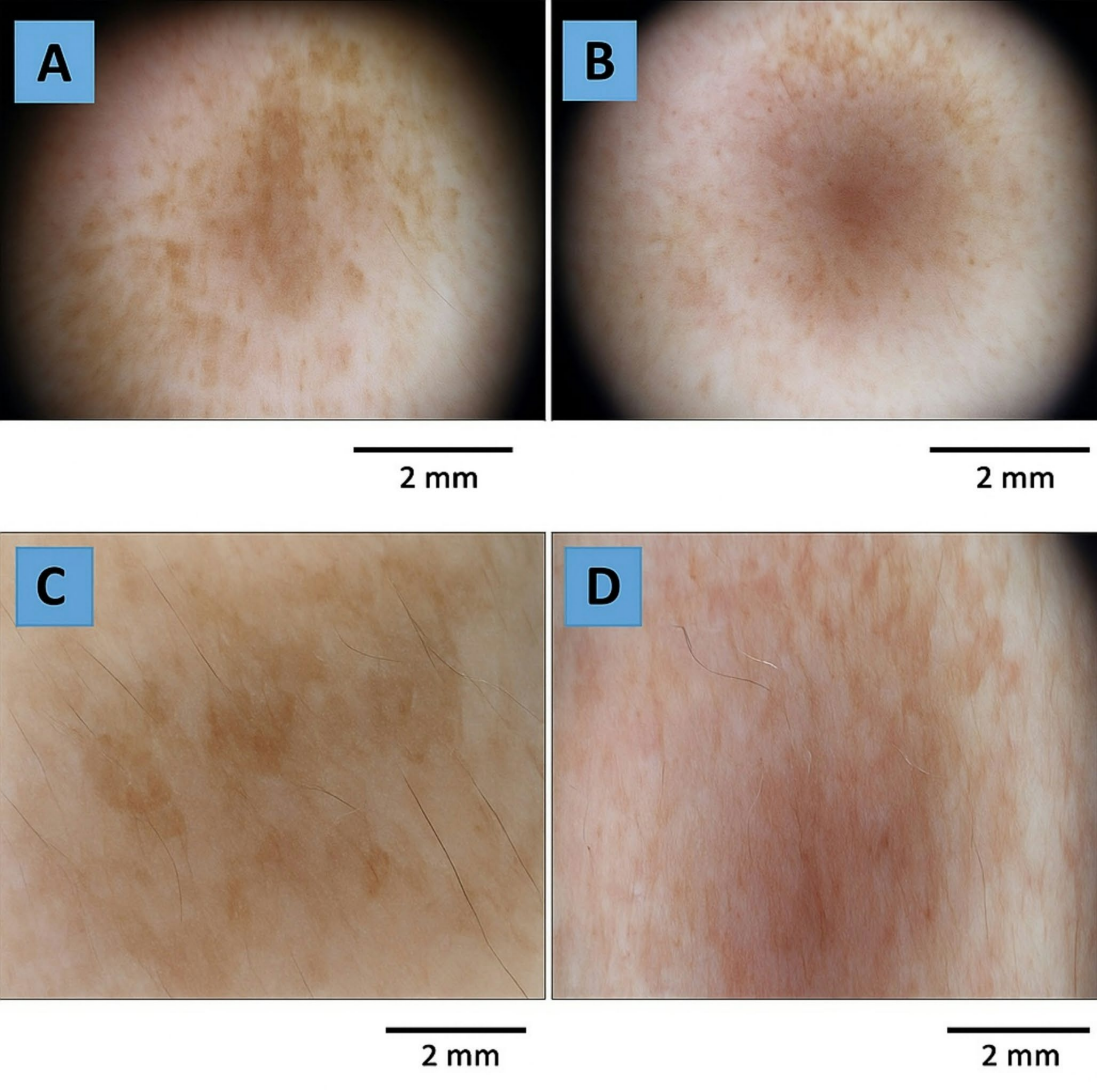
Dermoscopic images (**A**) Right side before treatment with tranexamic acid. (**B**) Right side after treatment showing fading of the pseudo-reticular pigment network. (**C**) Left side before treatment with ascorbic acid. (**D**) Left side after treatment with reduced telangiectasia and pigment intensity. Scale bar = 1 mm

**Fig. 3:**
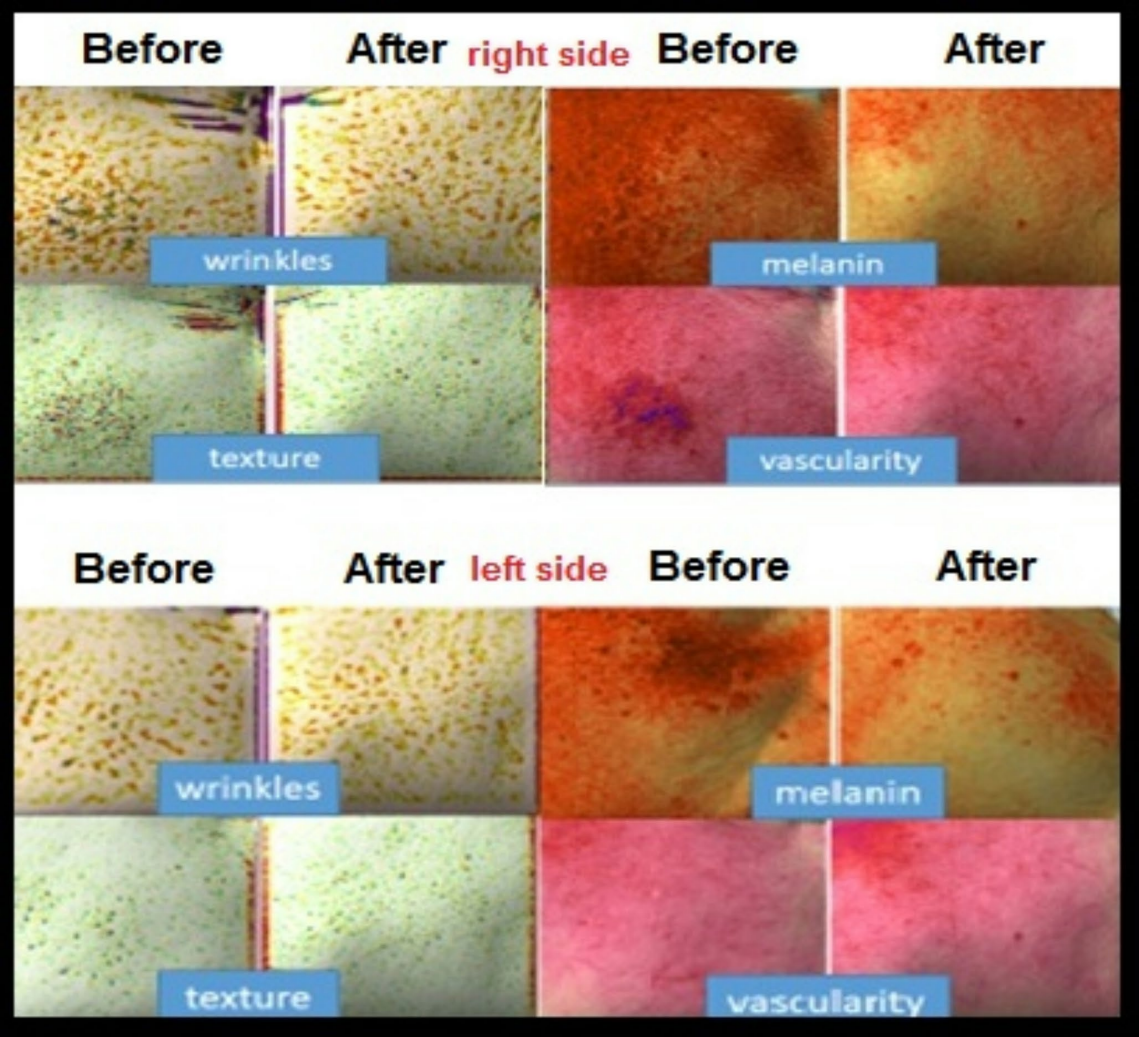
Antera (skin digital analysis) changes before and after treatment on the right side of the face (tranexamic treated side) and left side of the face (ascorbic acid treated side)

This study was conducted on 40 patients with melasma. Females outnumbered males in the study with 38 (95%) females and 2 (5%) male participants. The mean age of patients was 39.65 ± 5.66 years with range between (27–48 years). The mean melasma duration 4.75 ± 2.21 years and ranged between 1 and 9 years. (70%) of the patients had mixed melasma, while 30% had epidermal melasma. The Fitzpatrick skin type distribution was: Type III (*n* = 12), Type IV (*n* = 24), and Type V (*n* = 4). Baseline dermoscopic examination of the right side showed that 62.5% of the patients had fading of pseudo reticular network, 22.5% had fading telangiectasia, while the other 15% had no change. Considering the left side, 55% had fading of pseudo reticular network, 40% had fading telangiectasia, while the other 5% had no change. Fading of telangiectasia was significantly higher in the right side when compared to the left side (*p* = 0.02). Tables 1, 2 and 3; Figs. 1, 2 and 3.

**Table 1:** Main clinical characteristics of the studied cases

Parameter	
Duration of the disease (years)	*n* = 40
mean ± SD	4.75 ± 2.21
median (range)	4.5 (1–9)
Wood examination, n (%)	
mixed melasma	28 (70%)
epidermal melasma	12 (30%)
Dermoscopic examination, n(%)	
Right-side	
no change (10–40%) fading of pseudo reticular network (10–20%) fading of telangiectasia	6 (15%) 25 (62.5%) 9 (22.5%)
Left-side	
no change (10–40%) fading of pseudo reticular network (10%) fading of telangiectasia	16 (40%) 22 (55%) 2 (5%)

**Table 2:** Baseline dermoscopic features of both sides

Dermoscopic feature	Rt-side	Lt-side	*p*-value
no change	6 (15%)	16 (40%)	p = 0.01* x2 = 5.1
fading of pseudo reticular network	25 (62.5%)	22 (55%)	*p* = 0.49 x2 = 0.46
fading of telangiectasia	9 (22.5%)	2 (5%)	p = 0.02* x2 = 5.1

**Table 3:** Baseline characteristics of the studied cases before treatment

Parameter		Rt-side (*n* = 40)	Lt-side (*n* = 40)	*p*-value
Melanin	mean ± SD	0.75 ± 0.06	0.76 ± 0.06	*p* = 0.45 t = 0.74
	median (range)	0.76 (0.63–0.87)	0.77 (0.64–0.85)	
Vascularity	mean ± SD	1.92 ± 0.28	1.76 ± 0.24	p = 0.007* t = 2.74
	median (range)	1.86 (1.39–2.49)	1.72 (1.28–2.22)	
Wrinkles	mean ± SD	11.39 ± 1.23	11.36 ± 1.17	*p* = 0.91 t = 0.11
	median (range)	11.35 (9.23–13.78)	11.1 (9.37–13.42)	
Texture	mean ± SD	9.69 ± 1.05	9.83 ± 1.08	*p* = 0.55 t = 0.59
	median (range)	9.72 (7.94–11.43)	9.81 (7.98–11.57)	
Masi score	mean ± SD	5.13 ± 1.03	5.18 ± 1.08	*p* = 0.83 t = 0.21
	median (range)	5.02 (3.78–7.12)	5.26 (3.76–7.23)	

Data were expressed as mean *± SD and* median (range). Student t-test was applied. Masi: melasma area and severity index

The baseline characterstics of melasma were comparable except for vascularity which was significantly higher in the right side (*p* = 0.007). Following treatment; mean melanin and hemi MASI scores were significantly more reduced in the right side (tranaxemic acid treated) than the left side (ascorbic acid treated) (*p* = 0.04 and *p* = 0.002, respectively). The clinical outcome of the right-side showed a significant reduction of melanin, vascularity and mean hemi MASI score following tranexamic acid treatment (*p* = 0.001, *p* = 0.01 and *p* = 0.009, respectively) while on the left side only the mean melanin score was significantly reduced (*p* = 0.02) despite of non significant reduction of both the mean hemi MASI score and vascularity. Table 4.

**Table 4:** Comparison of clinical and instrumental outcomes before and after treatment on both sides of the face (right: tranexamic acid; left: ascorbic acid)

Parameter	Right Before (Mean ± SD)	Right After (Mean ± SD)	*p*-value (Right)	Left Before (Mean ± SD)	Left After (Mean ± SD)	*p*-value (Left)
Melanin	0.75 ± 0.06	0.70 ± 0.07	0.001	0.76 ± 0.06	0.73 ± 0.06	0.02
Vascularity	1.92 ± 0.28	1.77 ± 0.24	0.01	1.76 ± 0.24	1.69 ± 0.24	0.19
Wrinkles	11.39 ± 1.23	11.00 ± 1.16	0.14	11.36 ± 1.17	10.95 ± 1.29	0.14
Texture	9.69 ± 1.05	9.29 ± 1.12	0.10	9.83 ± 1.08	9.54 ± 1.05	0.22
MASI Score	5.13 ± 1.03	4.51 ± 1.06	0.009	5.18 ± 1.08	4.75 ± 1.11	0.08

Abbreviations: MASI, Melasma Area and Severity Index; SD, Standard Deviation. Statistical significance assessed using paired Student’s t-test

## Discussion

Despite the advances with technology and new formulations of medications, melasma remains challenging to treat. This study aimed to evaluate the efficacy of combined fractional CO2 laser with topical tranexamic acid versus combined fractional CO2 laser with topical ascorbic acid in treating patients with melasma. Among the study participants (*n* = 40), the majority were female (95%), with ages ranging from 27 to 48 years and an average age of 39.65 ± 5.66 years. The duration of melasma varied from 1 to 9 years, with an average of 4.75 years. Wood’s lamp examination identified the mixed pattern as the most prevalent (70%), followed by the epidermal pattern (30%). While the proportion of epidermal cases aligns with previous research, our study noted a higher occurrence of mixed melasma and no dermal cases [7]. 

A network meta-analysis of randomized controlled trials, which included 2,812 participants from 59 studies, reported that only 7.1% of subjects were males. The average participant age was 39.25 ± 6.69 years. Among identified melasma cases, the epidermal form was the most common (*n* = 280), followed by the mixed type (*n* = 105) and the dermal type (*n* = 57) [8].

Baseline dermoscopic evaluation of the right side revealed that 62.5% of patients experienced fading of the pseudo-reticular network, 22.5% had fading telangiectasia, and 15% showed no change. On the left side, 55% had fading of the pseudo-reticular network, and 40% showed fading telangiectasia. These results align with previous studies, which reported a prevalence of reticular pigment network in melasma patients ranging from 36–40% [9–11]. Differences in melanosome size and melanin density among various ethnic groups may explain the variation in pigmentation intensity. Additionally, telangiectasia has been identified as a characteristic of melasma due to UV-induced upregulation of vascular endothelial growth factor (VEGF), which is associated with melanogenesis [11]. 

Following treatment, melanin levels and hemi MASI scores showed a more significant reduction on the right side (treated with tranexamic acid) compared to the left side (treated with ascorbic acid) (*p* = 0.04 and *p* = 0.002, respectively). On the right side, a significant decrease in melanin, vascularity, and mean hemi MASI scores was observed (*p* = 0.001, *p* = 0.01, and *p* = 0.009, respectively). In contrast, on the left side, only melanin levels showed a significant reduction (*p* = 0.02), while changes in the mean hemi MASI score and vascularity were not statistically significant. Baseline imbalance in vascularity may have confounded interpretation of vascular improvement seen with TA on the left side.

Tranexamic acid has gained popularity in treating pigmentary disorders. However, there is no consensus regarding the optimal oral or topical dosage for melasma treatment [12]. Several studies have investigated topical tranexamic acid, with findings suggesting that while differences between topical tranexamic acid and placebo treatments were not statistically significant, the medication still led to a noticeable reduction in pigmentation compared to baseline data [13, 14].

A study evaluating the safety and efficacy of a 2% topical tranexamic acid emulsion found significant improvement after 18 weeks of treatment [15]. The combination of intense pulsed light (IPL) therapy with topical tranexamic acid was also shown to effectively reduce melasma and prevent recurrence [16]. Another study comparing topical tranexamic acid to a combination of hydroquinone and dexamethasone demonstrated a substantial decrease in MASI scores for both treatment groups [14]. 

Research conducted by Lee et al. indicated significant MASI score improvements following weekly intradermal injections of tranexamic acid [17]. Similarly, Budamakuntla et al. compared the effectiveness of microneedling-assisted topical tranexamic acid with tranexamic acid microinjections, finding a greater MASI score reduction in the microneedling group, although the difference was not statistically significant [18]. In Egypt, a study comparing microneedling with tranexamic acid to 4% hydroquinone treatment reported enhanced response rates with the microneedling approach and no adverse effects [19]. 

Antera 3D; a multi- led handheld camera with accompanying software captured three-dimensional high resolution multimode images that allowed to quantify the efficacy of treatments and to map and monitor changes over time in terms of topography, roughness index, indentations index, average melanin level, and hemoglobin. A study compared the therapeutic efficacy and safety of TA 5% vs. HQ 4% showed significant improvement of different types of melasma without significant difference regarding melasma scores and average levels of melanin when measured by Antera imaging [20]. 

Ascorbic acid (vitamin C) is a well-documented antioxidant that inhibits melanogenesis by binding to copper and blocking tyrosinase activity. However, its trans-epidermal penetration is limited. A split-face trial using ascorbic acid serum showed improvement in 15% of melasma patients, with a more pronounced effect when combined with microneedling [21]. When compared to 4% hydroquinone, ascorbic acid treatment exhibited slower but noticeable improvement, becoming evident after three months, whereas hydroquinone showed more rapid effects within the first month [22]. 

A study investigating intradermal injections of combined tranexamic acid and ascorbic acid found that MASI scores significantly decreased, with improvements sustained for three months [23]. Additionally, microneedling combined with topical vitamin C has been identified as an effective and safe treatment for melasma in individuals with Fitzpatrick skin types III-IV [24]. Other studies have explored microneedling to enhance vitamin C penetration, and a pilot study on Middle Eastern women suggested that a combination of 2% tranexamic acid and 2% vitamin C is a good option for treating resistant melasma [25].– [26].

One study compared tranexamic acid microinjection alone to its combination with low-power, low-density fractional CO2 laser treatment, finding both approaches effective but recommending the combined approach for better outcomes [27]. Another study assessed the use of microneedling versus fractional CO2 laser for tranexamic acid delivery, concluding that both techniques were safe and effective for treating facial melasma [28]. 

Limitations to the study were the relatively smaller sample size of included subjects. In addition, selection bias, as the patients were primarily recruited from patients who attended one university clinic only. Although no PIH was observed, the use of fractional CO_2_ laser in Fitzpatrick skin types III–V carries a known risk. Our conservative laser settings were selected to minimize this, but longer follow-up is warranted to monitor for late-onset PIH. A vehicle-only or placebo control was not used. Both agents were applied under identical occlusion conditions; however, differences in formulation could have affected outcomes. Additionally, although the sample size provided adequate power for detecting moderate treatment effects, no formal correction for multiple comparisons was applied. Utilizing a multicenter approach would improve generalizability of the results, a larger sample size, and, consequently, improved efficiency. Another limitation was the short follow up period.

In conclusion, the trans-epidermal delivery of tranexamic acid or vitamin C following low-power fractional CO2 laser treatment appears to be a beneficial approach for melasma management, with tranexamic acid demonstrating superior efficacy. Further large-scale controlled trials are needed to determine the most effective and standardized treatment regimen for melasma.

## Data Availability

The data that support the findings of this study are available from the corresponding author upon reasonable request.

## Code availability

All authors confirm all data and materials as well as software applications or custom code support their published claims and comply with field material

## Abbreviations

AFR: Ablative Fractional Resurfacing
bFGF: Basic Fibroblast Growth Factor
FGF-2: Fibroblast Growth Factor 2
FP: Fractional Photothermolysis
MASI: Melasma Area and Severity Index
mMASI: modified MASI
SPSS: Statistical Package for the Social Sciences
TA: Tranexamic Acid
UV: Ultraviolet
VEGF: Vascular Endothelial Growth Factor
